# Supercritical CO_2_ Extraction of Fatty Acids, Phytosterols, and Volatiles from Myrtle (*Myrtus communis* L.) Fruit

**DOI:** 10.3390/molecules29081755

**Published:** 2024-04-12

**Authors:** Daniela Cvitković, Iva Škarica, Verica Dragović-Uzelac, Sandra Balbino

**Affiliations:** Faculty of Food Technology and Biotechnology, University of Zagreb Pierottijeva 6, 10000 Zagreb, Croatia; dcvitkovic45@gmail.com (D.C.); iva.skarica96@gmail.com (I.Š.); vdragov@pbf.hr (V.D.-U.)

**Keywords:** myrtle, supercritical fluid extraction, fatty acids, sterols, volatiles

## Abstract

Background: Myrtle (*Myrtus communis* L.) is a coastal Mediterranean aromatic medicinal plant rich in essential oil components, flavonoids, and phenolic acids. Studies highlight the potential health benefits of myrtle bioactive compounds with antioxidant and antiproliferative properties. Since limited research exists on myrtle fruit’s lipid fraction, the aim of this study was to apply supercritical CO_2_ extraction to obtain bioactive compounds from myrtle berries focusing on the fatty acids, sterols, and essential oils. Methods: The optimization of the supercritical CO_2_ extraction of myrtle fruit using CO_2_ as solvent was carried out using the response surface methodology with Box–Behnken experimental design. The following conditions were tested: temperature (40, 50, and 60 °C), pressure (200, 300, and 400 bar), and flow rate (20, 30, and 40 g min^−1^) on the yield of lipid extract as well as on the yield of fatty acids, phytosterols, and volatiles present in the extract and constituting its bioactive potential. Results: In the extracts examined, 36 fatty acids, 7 phytosterols, and 13 volatiles were identified. The average yield of the extract was 5.20%, the most abundant identified fatty acid was essential cis-linolenic acid (76.83%), almost 90% of the total phytosterols were β-sitosterol (12,465 mg kg^−1^), while myrtenyl acetate (4297 mg kg^−1^) was the most represented volatile compound. The optimal process conditions obtained allow the formulation of extracts with specific compositions.

## 1. Introduction

Myrtle (*Myrtus communis* L., Myrtaceae) is an aromatic medicinal plant typical of the coastal Mediterranean areas, such as North Africa or Southern Europe, and is also found in South America, Australia, and certain areas of the Himalayas [1]. This small tree or shrub, which grows about 2 m tall, has small aromatic evergreen leaves, fragrant white or rosy flowers, and blue-black fruits rich in seeds which ripen between October and February [2,3]. Myrtle leaves are rich in essential oil components, flavonoids, and phenolic acids most commonly used in the perfume industry, while the fruits contain essential oil, anthocyanins, tannins, and fatty and organic acids [4]. Myrtle fruits are mainly used in the food sector for traditional Italian liqueur production, especially on the island of Sardinia, which has a geographical indication of origin, known as “Mirto di Sardegna” [2], with an annual production of more than three million bottles [5], as well as for flavoring meat and sauces [6]. 

Supercritical fluid extraction (SFE) is an environmentally friendly extraction technique that uses supercritical fluids with liquid and gas-like properties at temperatures and pressures above the critical point. Unlike the most commonly used solvents for oil extraction such as hexane, methanol, and chloroform, these solvents are gases at ambient conditions and are usually non-toxic [7]. There is a wide range of solvents that can be used in supercritical fluid extraction: nitrogen, methane, ethylene, xenon, etc. Due to a number of positive properties related to carbon dioxide as a solvent, such as low cost, safety, non-toxicity, non-flammability, GRAS status, and the ability to operate at room temperature and low pressure, it is the most common choice in SFE. Also, the product obtained is solvent-free due to the gaseous state of CO_2_ [8]. CO_2_ as a fluid has a critical temperature (31.1 °C) and critical pressure (7.38 MPa), and above these values it becomes supercritical; therefore, adjusting the temperature and pressure values affects its extractability and changes its properties such as density, viscosity, heat capacity, diffusivity, etc. [9]. Supercritical CO_2_ (scCO_2_) is an excellent choice for the extraction of thermolabile nonpolar compounds such as triglyacylglycerols, terpenoids, fatty alcohols, phytosterols, and tocopherols [10]. When designing and optimizing the scCO_2_ extraction process, the parameters applied during extraction must be adjusted, i.e., pressure, extraction time, temperature, solvent flow rate, and the use of co-solvents. Likewise, the particle size of the material to be extracted is of great importance, the reduction of which has a positive effect on the extraction yield (King, 2014) [11]. The efficacy of scCO_2_ extraction on myrtle fruit has been investigated in only two studies focused on the essential oil [12] and antioxidant capacity [13], while no studies are available for the lipid fraction of myrtle fruit. In addition, the composition of phytosterols as valuable bioactive compounds that may be present in myrtle fruit has not yet been studied.

Fatty acids are carboxylic acids whose hydrocarbon chain can be saturated or unsaturated, containing one (monounsaturated) or several (polyunsaturated) double bonds. While saturated and trans fatty acids are known for their harmful effects on health, polyunsaturated fatty acids show beneficial effects, such as reducing cardiovascular and coronary heart diseases, by lowering serum cholesterol levels, blood pressure, arrhythmia, etc. [14]. Polyunsaturated fatty acids (PUFAs) such as linoleic and linolenic fatty acid are essential to the human body, and their metabolites are precursors of important cellular processes such as the inflammatory response [15]. According to different authors, the total proportion of oil in the fruits of different myrtle genotypes ranges from 2.73% [16] to 8.90% [17], while increasing maturity increases the total content of fatty acids in the fruits [18]. The content of unsaturated fatty acids in the myrtle fruit is about 84%, most of which are polyunsaturated fatty acids (PUFAs), while saturated fatty acids account for about 16% [5]. The most abundant unsaturated fatty acids in the myrtle berries are oleic as monounsaturated and linoleic as polyunsaturated, while palmitic and stearic are major saturated fatty acids [6].

Phytosterols are phytosteroids found in the membranes of plant cells and include plant sterols, which are triterpene analogs of cholesterol, and stanols, which have a saturated ring structure (Younas et al., 2023) [19]. The most abundant sterols in foods are campesterol, sitosterol, and stigmasterol, while campestanol and sitostanol are the most abundant sterols in the stanols group [20]. The positive effects of phytosterols on human health are well known, such as the protective effect on cardiovascular diseases, diabetes, and tumors [21]. 

Essential oils are fragrant, volatile compounds that contain many different constituents and are stored in the secretory structures of plants, such as glands. Their proportion rarely exceeds 1% of the total mass of the plant [22]. The compounds that make up the essential oils of myrtle can be divided into terpenes (monoterpene hydrocarbons and sesquiterpene hydrocarbons), terpenoids (oxygenated monoterpenes and oxygenated sesquiterpenes), phenylpropanoids, hydrocarbons, and oxygenated compounds [4]. Various biological effects such as antiproliferative and antioxidant activity are attributed to the chemical compounds of myrtle essential oil [23]. In their study, Jerkovic et al. [24] determined the most abundant terpenoids in myrtle fruit essential oil: myrtenyl acetate, 1,8-cineole + limonene, α-pinene, and linalool.

In this study, the bioactive potential of myrtle fruit extracts, with an emphasis on lipophilic compounds (fatty acids, phytosterols, and volatile compounds), and the influence of supercritical CO_2_ extraction conditions (temperature, pressure, and flow rate) on the yield of the extracts and compounds studied are investigated. Likewise, the suitability of the response surface methodology with the Box–Behnken experimental design to optimize this extraction is investigated.

## 2. Results and Discussion

### 2.1. Extraction Yield

Table 1 shows the experimental design with the conditions used in each experiment and the obtained yields of scCO_2_ extracts. The average yield of lipophilic myrtle fruit extract obtained in the experiments according to the Box–Behnken experimental design is 5.20% and ranges from 4.75 to 5.92%. The lowest extraction yield was obtained under the experimental conditions of a temperature of 40 °C, a pressure of 200 bar, and a flow rate of 30 g min^−1^, and the highest at a temperature of 60 °C, a pressure of 300 bar, and a flow rate of 40 g min^−1^. The yields of scCO_2_ extracts of myrtle fruit reported by Pereira et al. [13] were significantly higher than in the present study, ranging from 8.8% to 14.1%. However, since the aforementioned study used ethanol as a co-solvent in supercritical extraction and focused on the determination of polyphenols, the higher yield may be attributed to the improved extraction of these compounds. Since other studies on the scCO_2_ extraction of myrtle have been performed with leaves, the results obtained in this study can be compared with the results of fruit extraction using conventional techniques and solvents. The yield of lipid fraction in the fruits of different myrtle genotypes determined using Soxhlet ranges from 3.83 to 5.90% [6,25], which is comparable to the results reported in the present study.

### 2.2. Fatty Acids, Phytosterols, and Volatiles in Myrtle Berries

Table 2 shows the results of fatty acid (saturated and unsaturated), phytosterol, and volatile compound determinations. The studied groups of compounds were determined using gas chromatography in samples of supercritical extracts from myrtle fruits.

#### 2.2.1. Fatty Acid Composition

The gas chromatographic analysis of FAME was able to identify 36 fatty acids (FAs), 17 saturated (SFAs), 9 monounsaturated (MUFAs), and 10 polyunsaturated fatty acids (PUFAs) (shown in Supplementary Materials). To maintain the clarity of presentation, Table 2 shows the average contents only for FAs present in average contents > 0.1%. Other FAs that were determined but are not included in the table were the following: C4:0, C6:0, C8:0, C10:0, C12:0, C13:0, C14:0, C21:0, C14:1, C15:1, C16:1, C17:1, C18:1t, C22:1, C24:1, C18:3n6, C20:2, C20:3n3, C22:2, and C22:6. The dominant FA in the analyzed extracts was essential cis-linoleic (C18:2c) FA, with an average content of 76.83%, followed by palmitic (C16:0) (8.52%), oleic (C18:1) (7.29%), and stearic FA (C18:0) (3.09%). The average content of SFAs is 14.3%, MUFAs are at 7.77%, while PUFAs are the most prevalent FA group (77.93%), which is mainly due to the high percentage of linoleic FA, while other PUFAs (α-linolenic (C18:3) and n-3-eicosapentaenoic FA (C20:5n3)) are present in contents lower than 0.50%. According to the study of Şan et al. [6], the main SFAs in myrtle fruits are palmitic (10.18–13.40%) and stearic (2.93–4.34%), oleic FA (10.14–13.48%) is the main MUFA, and linoleic FA (69.47–71.71%) is the most dominant polyunsaturated FA. A slightly lower range was found by Özcan et al. [26], who reported the proportion of oleic acid to be 7.47–9.21%, which is almost identical to the results of the present study. Similar values, in accordance with our results, were determined by other authors [25,26]. In contrast, significant deviations in FA composition from the composition determined in this work were found by [27] for linoleic (4.78%) and oleic FA (67.07%). In the study of [28], oleic was the most abundant FA and almost ten times higher than reported in the present study (64.1–72.1%). This wide variation in the composition of the FAs could be the result of different myrtle fruit harvesting times, i.e., different stages of ripeness of the myrtle fruit, the significant influence of which was demonstrated by Wannes et al. [18]. As the fruit ripened, the content of oleic FA significantly decreased (from 21.89 to 6.46%), while the content of linoleic FA significantly increased (from 12.21 to 71.34%).

#### 2.2.2. Phytosterols Composition

As mentioned, this is the first publication of data on the composition of phytosterols in myrtle fruit. Seven phytosterols were identified in SF extracts of myrtle fruit: campesterol, 24-methylenecholesterol, stigmasterol, β-sitosterol, Δ5-avenasterol, Δ7-sitosterol, and citrostadienol (shown in Supplementary Materials). The average content of total phytosterols in the extracts was 13,939 mg kg^−1^, with β-sitosterol accounting for 90%, followed by Δ5-avenasterol and campesterol. Shen et al. [29] compared the contents of individual and total phytosterols in most commercial vegetable oils such as palm, soybean, corn germ, rapeseed, olive, etc. They reported the highest content of total phytosterols in corn germ and rapeseed oil in the range of 6128.7–9525.5 mg kg^−1^ and 5917.0–6858.9 mg kg^−1^, respectively. In their study, Vecka et al. [30] found the highest phytosterol content in sesame (6590 mg kg^−1^) and pistachio (5850 mg kg^−1^) among lipid extracts isolated from 19 different species of seeds, nuts, and kernels. A comparison with the data from our studies shows that extracts from myrtle fruits have, on average, twice as much phytosterol content than the above-mentioned oils. In most of them, β-sitosterol was dominant, followed by campesterol and stigmasterol. According to Moghadasian [31], sitosterol and campesterol account for about 95% of the phytosterols present in food, while the remaining 5% consists of other sterols or stanols, mainly stigmasterol. The average yield of phytosterols was 723 mg per kg^−1^ of myrtle fruit, which, compared to the results of other plant species, also confirms that myrtle fruit is a rich source of phytosterols. Balbino et al. [32] analyzed the amount of phytosterols in extracts of fennel, anise, caraway, and coriander and found that the amount of phytosterols ranged from 390.3 to 542.6 mg per kg^−1^ seed. In another study, the content of phytosterols in sea buckthorn fruits was found to be in the range of 344–515 mg per kg^−1^ fruits [33].

#### 2.2.3. Volatile Composition

Total volatiles identified in myrtle fruit extracts averaged to 9020 mg kg^−1^ with dominant compounds being myrtenyl acetate (47.6%), 1,8-cineole (24.2%), and α-pinene (14.2%) (shown in Supplementary Materials). Given the high content of myrtenyl acetate, the myrtle used for the experiments in this study belongs to the myrtenyl acetate chemotype, the occurrence of which is characteristic of the countries of the former Yugoslavia, as well for Portugal, France, etc., as opposed to the 1,8-cineol chemotype found in Algeria and Tunisia [34]. These results can be compared to the studies of Pereira et al. [35] and Jerkovic et al. [24], in which the most common compounds identified in myrtle fruit essential oil are myrtenyl acetate, 1,8-cineole, limonene, α-pinene, and linalool. In these studies, their ranges were 12.2–33.2% and 32.86–36.48% for myrtenyl acetate, 10.9–21.1% and 21.02–25.28% for 1,8-cineole + limonene, 4.0–15.3% and 4.08–9.65% for α-pinene, and 4.7–7.7% and 6.56–7.50% for linalool, respectively. Ghasemi et al. [36] found that the content and composition of volatiles obtained by means of scCO_2_ differ from those obtained by means of hydrodistillation. In their work, 17 compounds were found in the scCO_2_ extract of myrtle compared to 31 compounds obtained by means of hydrodistillation. The average extraction yield of volatile compounds is 465.91 mg kg^−1^ of fruit, which is comparable to the results of Usai et al. [37] obtained by means of hydrodistillation, in which 550 mg kg^−1^ was the highest yield among 47 different myrtle cultivars and the majority was below 100 mg kg^−1^.

### 2.3. Influence of SFE Conditions

The RSM method was used to study the influence of each scCO_2_ factor and their interactions. Considering that many factors such as temperature, pressure, extraction time, solvent flow rate, particle size, water content, and the use of co-solvents affect the yield of target components, the modelling of the process parameters is essential for the development of an effective extraction method for obtaining extracts from plant materials rich in bioactive molecules. RSM is a suitable platform for optimizing the extraction and developing a process that is efficient from both an economic and a production perspective [38]. The independent variables tested were extraction yield, PUFA content, and phytosterol and volatile compound yield. For each of the selected dependent variables, three models were compared, namely, linear, two-factor interaction, and quadratic, while statistical parameters (*p*-value, lack of fit, and R^2^) were used to select those that best describe the relationship between independent and dependent variables.

#### 2.3.1. Extraction Yield

For extraction yield, a linear model (*p* < 0.001) with a coefficient of determination R^2^ = 0.799 was chosen, while the lack of fit was not significant (*p* = 0.164). Extraction yield was significantly influenced by temperature (*p* < 0.05), while the influence of the other two factors was not significant. The regression coefficients of the model show that extraction yield is increased by 0.036 % for each degree rise in temperature.

Extraction yield (%) = 3.036 + 0.036 × Temperature + 5.250 × 10^−4^ × Pressure + 7.624 × 10^−3^ × Flow rate (1)

**Figure 1:**
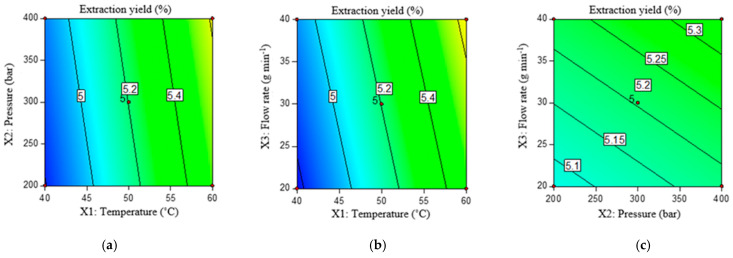
Contour plots of extraction yield (%) under the influence of temperature (°C) and pressure (bar) (**a**), temperature (°C) and flow rate (g min^−1^) (**b**), and pressure (bar) and flow rate (g min^−1^) (**c**).

The same is evident from Figure 1, which shows the contour plots of the influence of the combination of independent scCO_2_ extraction factors on the lipophilic extract yield. The plots were made by varying two factors in the experimental range, while the third variable was set to the value of the central point. 

Temperature is one of the most important factors in the extraction of lipophilic compounds by means of scCO_2_, as temperature changes affect their solubility. For the extraction of seed oil, temperatures in the range of 40 to 80 °C are usually used [11]. In the Gustinelli et al. [39] study, increasing the temperature and pressure from 50 °C and 20 MPa to 60 °C and 50 MPa increased the yield of bilberry seed oil from 7.6% to 22.2%. The temperature has a two-fold effect on the extraction yield: its increase affects the reduction in the density of the supercritical fluid, which reduces its extraction efficiency, and on the other hand, it increases the volatility of the compounds and thus improves the mass transfer and solubility of the components [40]. Similar to these results, in the study by Teixeira et al. [41], in which the influence of pressure and temperature on the yield in the extraction of sapucaia nut oil was investigated, only temperature had a significant influence.

#### 2.3.2. Polyunsaturated Fatty Acid Yield

**Figure 2:**
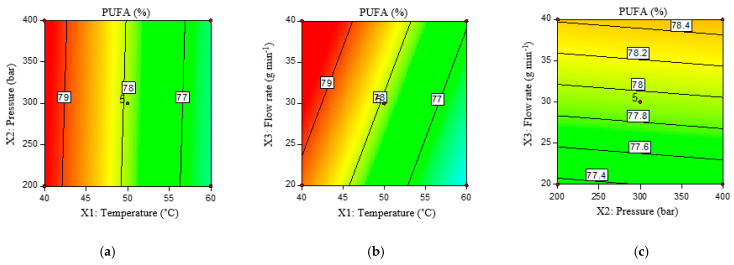
Contour plots of PUFA yield (%) under the influence of temperature (°C) and pressure (bar) (**a**), temperature (°C) and flow rate (g min^−1^) (**b**), and pressure (bar) and flow rate (g min^−1^) (**c**).

To show the influence of scCO_2_ conditions on the content of total PUFAs, a linear model was chosen as the most appropriate. The significance of the model was *p* = 0.008, with a coefficient of determination R^2^ = 0.587, while the lack of fit was not significant (*p* = 0.095), and the contour plots of the model are shown in Figure 2.

PUFA (%) = 83.264 − 0.141 × Temperature + 4.141 × 10^−4^ × Pressure + 0.053 × Flow rate (2)

From Figure 2 and Table 3, as well as from the model itself, it is evident that temperature is the only significant factor, with the content of PUFAs decreasing as temperature increases. The reason for this could be the different solubility of the fatty acids at different scCO_2_ temperatures and pressures. In accordance with our results, in the study by Jokić et al. [42], temperature (40–60 °C) had a more significant effect on the composition of fatty acids than pressure (300–500 bar), the increase in which affected the reduction of linolenic and linoleic fatty acids as the most abundant PUFA in soybean oil. However, in the study by Teslić et al. [43] on wheat germ oil extraction, it was found that pressure and CO_2_ flow had the greatest influence on PUFA content, while the influence of temperature was quite weak. According to research by Maheshwari et al. [44] conducted on lauric, myristic, palmitic, stearic, oleic, and linoleic fatty acids, their solubility depends not only on temperature but also on the applied CO_2_ pressure. At a temperature of 40 °C, the solubility of linoleic fatty acid in CO_2_ increases from 45 to 220 g g^−1^ CO_2_ with a pressure increase from 138 to 207 bar, while at 60 °C, this increase ranges from 11 to 180 g g^−1^ CO_2_. At a further pressure increase up to 276 bar, the increase in solubility is smaller, i.e., it is not significant. It is therefore likely that the influence of pressure was not significant in our study, since the lowest pressure tested was 200 bar.

#### 2.3.3. Phytosterols Yield

The quadratic model shown in Equation (3) (*p* = 0.024; R^2^ = 0.864; and lack of fit *p* = 0.164) best describes the influence of factors on phytosterol yield (mg kg^−1^).

Phytosterols yield (mg kg^−1^) = 296.411 − 29.628 × Temperature + 3.787 × Pressure + 39.402 × Flow rate + 0.029 × Temperature × Pressure + 0.064 × Temperature × Flow rate − 0.072 × Pressure × Flow rate + 0.170 × Temperature^2^ − 0.005 × Pressure^2^ − 0.300 × Flow rate^2^ (3)

**Figure 3:**
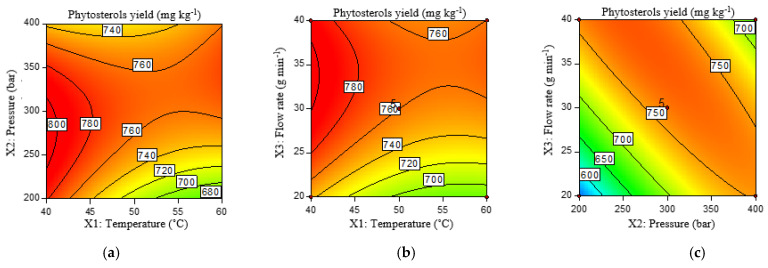
Contour plots of phytosterols yield (mg kg^−1^) under the influence of temperature (°C) and pressure (bar) (**a**), temperature (°C) and flow rate (g min^−1^) (**b**), and pressure (bar) and flow rate (g min^−1^) (**c**).

From the model and contour plots in Figure 3 and Table 3, it can be seen that increasing pressure and flow rate increases the yield of phytosterols regardless of the applied temperature, with a significant linear component of flow rate and a quadratic component of pressure. The effect of temperature is not significant, while the interaction of pressure and flow rate is significant among the other factors of the quadratic model. In fact, it is observed that the content of phytosterols increases with increasing pressure at a lower flow rate, while it decreases with increasing pressure at a higher flow rate. Generally, the flow rate of the solvent must be high enough to ensure a good extraction yield in a short time, but it must also allow sufficient contact time between the solvent and the solutes [45]. Increasing the flow rate leads to a shorter contact time, which reduces mass transfer [46]. In addition, increasing the flow rate leads to an increase in the intermolecular interactions between the CO_2_ and the target molecules. Thus, from the results of this study, the positive influence of increased flow predominates at lower pressures, while it is negative at higher pressures. A decrease in β-carotene yield from rosehip fruit at a flow rate greater than 3 mL min^−1^ was also observed by Machmudah et al. [47], and similarly described using the example of lycopene extraction from tomato by Zuknik et al. [48]. In their study on the effects of SFE on lotus bee pollen, Xu et al. (2010) found that a higher temperature at a low pressure resulted in lower yields of phytosterols, which is explained by a stronger effect of temperature increase on the decrease in CO_2_ density than on the vapor pressure of the solute. Similar conclusions were reached by Grzegorz et al. [49], who investigated the proportion of phytosterols in SF flaxseed extracts. However, in our study, the effect of temperature on the yield of phytosterols from myrtle fruits was not significant, supporting the fact that the effects of scCO_2_ conditions strongly depend on the interaction with the matrix, i.e., they differ depending on the properties of the material to be extracted [50].

#### 2.3.4. Volatiles Yield

The influence of the parameters used in this experiment is best described by the quadratic model shown in Equation (4) (*p* = 0.018; R^2^ = 0.876; and lack of fit *p* = 0.710):

Volatiles yield (mg kg^−1^) = −2090.814 + 114.555 × Temperature + 4.920 × Pressure − 51.654 × Flow rate − 0.013 × Temperature × Pressure + 0.189 × Temperature × Flow rate + 0.017 × Pressure × Flow rate − 1.230 × Temperature^2^ − 0.008 × Pressure^2^ + 0.553 × Flow rate^2^ (4)

**Figure 4:**
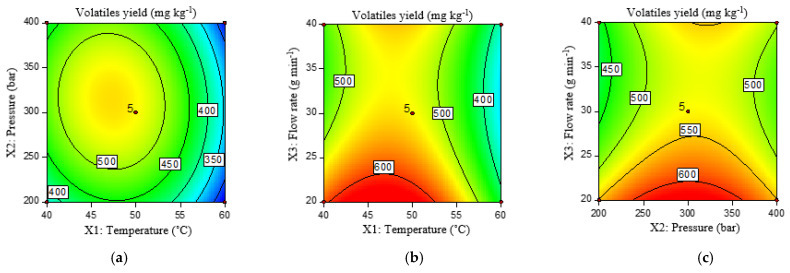
Contour plots of volatile yield (mg kg^−1^) under the influence of temperature (°C) and pressure (bar) (**a**), temperature (°C) and flow rate (g min^−1^) (**b**), and pressure (bar) and flow rate (g min^−1^) (**c**).

The linear and quadratic component of temperature and the quadratic component of pressure significantly affected the yield of volatile compounds. From the above results (Figure 4), it can be seen that the highest yield of volatile compounds is obtained at a moderate pressure and temperature values while at higher temperatures, the yield decreases. The interaction between flow rate (g min^−1^) and temperature (°C) on the yield of volatile compounds had no significant effect. Most volatiles are obtained by increasing the temperature to certain values (about 47 °C), while further increasing the temperature decreases the yield of volatile compounds regardless of the applied flow rate. The effect of pressure is similar, with the maximum yield of volatiles obtained at a pressure of about 300 bar, i.e., near the central point.

Similar results describing the significant influence of pressure and temperature on volatile compounds were also found in the studies of other authors. For example, Zhao and Zhang [51] showed that an increase in temperature from 40 °C to 60 °C resulted in a significant increase in the extraction yield of Moringa oleifera plant leaves, while a further increase in temperature to 80 °C did not cause a significant increase. Since an increase in temperature leads to a decrease in the density of the solvent on one hand and increases the vapor pressure and solubility of the volatile compounds on the other, the yield of volatile compounds depends on a balance between the scCO_2_ density and the volatility of the compounds. Grosso et al. [52] also concluded in their study that pressure and temperature affect the yield and composition of volatiles. However, if the pressure is increased at the same temperature, the non-volatiles also dissolve. Increasing the temperature at the same pressure leads to a decrease in the density of the solvent, resulting in a lower yield and an oil with a higher content of monoterpene hydrocarbons, which is why moderate temperature and pressure conditions are recommended. A similar influence of pressure and temperature and their moderate values as optimal for the extraction of volatile compounds from fennel were also confirmed by Maitusong et al. [53].

### 2.4. Results of the Optimization of SFE Conditions

A practical disadvantage of SFE is the large number of factors that can affect the results and should be examined before starting a larger scale production. Therefore, in order to rationalize the number of experiments, an experimental design using RSM is often used. The Box–Behnken experimental design used in this study is one of the most common tools for optimizing SFE conditions [54]. As evident from various papers dealing with the determination of optimal scCO_2_ extraction conditions [55], optimal temperature, pressure, and CO_2_ flow significantly depend on the matrix and chemical composition of the extracted compounds. Considering the nonlinearity of the SFE system and the observed differences in the influence of temperature, pressure, and flow rate on the individual target components, the optimization of the extraction conditions was performed separately for the lipophilic extract, PUFA, sterol, and volatile yield of the myrtle fruits using the desirability method. These parameters were marked with the importance factor set to 5 in each optimization run. In this way, it was possible to recommend the process parameters required for obtaining extracts with specific compositions of bioactive components and for specific purposes.

Table 4 shows the optimal conditions for achieving the maximum values of the above dependent variables. To achieve the maximum extraction yield of 5.69%, extraction conditions must be applied at the highest level of the tested range, i.e., a temperature of 60.0 °C, a pressure of 400.0 bar, and a flow rate of 40 g min^−1^. Mendiola et al. [45] state that the optimal conditions for the SFE of bioactive compounds from Spirulina are achieved at the maximum pressure and temperature tested, i.e., 83.1 °C and 362 bar, resulting in the maximum extraction yield of 0.53%. Furthermore, the maximum PUFA yield of 79.34% requires much milder extraction conditions, which include a temperature of 40.2 °C, a pressure of 235.7 bar, and a flow rate of 31.1 g min^−1^. In their study, Teslić et al. [43] performed the SFE of wheat germ under the following conditions: a pressure of 250–350 bar, a temperature of 40–60 °C, and a flow rate of 0.2–0.4 kg h^−1^. The maximum PUFA content of 7.25% was obtained at a pressure of 350 bar, a temperature of 50 °C, and a flow rate of 0.4 kg h^−1^ (6.67 g min^−1^). It can be seen that the maximum values for pressure and flow rate as well as the average value for temperature were used to achieve the maximum PUFA content, which differs from the results of our research, but it should be taken into account that this is a different plant matrix [50]. Very similar extraction conditions determined in this study for PUFA extraction are also optimal for phytosterol extraction, being 40.5 °C, 225.0 bar, and a flow rate of 32.5 g min^−1^. Using the above conditions, it is possible to obtain sterols yield of 793.48 mg per kg^−1^ myrtle fruit. In the study by Sajfrtová et al. [56], scCO_2_ extraction was performed on sea buckthorn at pressures of 150–600 bar and temperatures of 40–80 °C. The maximum concentration of β-sitosterol in the extract was obtained at a pressure of 150 bar and a temperature of 40 °C, which agrees with our results, since a higher yield of phytosterols is obtained at moderate conditions. The optimum scCO_2_ extraction conditions for volatiles are 49.5 °C, 298.0 bar, and 20.3 g min^−1^, i.e., for the maximum yield of volatiles determined in this work, which is 629.85 mg kg^−1^, moderate values of pressure and temperature and the lowest flow rate tested are required. In a study by Pereira et al. [13], a temperature of 45 °C, a pressure of 230 bar, and a CO_2_ flow rate of 5 g min^−1^ were used as conditions for the extraction of essential oil from myrtle fruit. These values were obtained in a previous study by the same group of authors, which involved the optimization of the SFE of myrtle leaves [57]. However, the plant parts used for extraction are not the same and the flow rate range is much lower in their case.

## 3. Materials and Methods

### 3.1. Chemicals

Commercial standards of myrtenyl acetate, 1,8-cineole, p-cymene, (+)-α-pinene, (−)-β-pinene, γ-terpinene, (R)-(−)-α-phellandrene, carvacrol, (+)-carvone, camphene, 3-carene, geraniol, o-cymene, and a mixture of α-fenchyl acetate and alkane standard solution C7-C30 were obtained from Sigma Aldrich (St. Louis, MO, USA); α-terpineol, myrcene, linalool, butyl butyrate, and eugenol from Merck (Darmstadt, Germany); R-(+)-limonene and nerol from Fluka^®^ Analytical (Munich, Germany); and α-terpinene from Dr. Ehrenstorfer GmbH (Augsburg, Germany). Reagents for silylation, α-cholestanol, campesterol, stigmasterol, and β-sitosterol were purchased from Sigma Aldrich (St. Louis, MO, USA). The standard mixture of 37 fatty acid methyl esters (Supelco™ 37 Component FAME Mix) was obtained from Sigma-Aldrich (St. Louis, MO, USA). Aluminum oxide, sodium hydroxide, sodium chloride, and sodium hydrogen sulfate monohydrate were procured from Merck KgaA (Darmstadt, Germany). Diethyl ether, anhydrous ethanol, ethyl acetate, and methanol were acquired from Carlo Erba Reagents GmbH (Emmendingen, Germany). Isooctane, isopropanol, potassium hydroxide, 96% ethanol, and n-hexane were obtained from J. T. Baker (Phillipsburg, NJ, USA).

### 3.2. Plant Material

Myrtle (*Myrtus communis* L.) fruits (1 kg), collected in February 2021 in the ecologically clean area of Mljet, were dried (Inkolab, Jumo, Zagreb, Croatia) at 45 °C for 18 h to a dry matter content of 96.23%. They were then ground in an electric laboratory mill (WSG30, Waring Commercial, Torrington, CN, USA) and sieved through a 1.5 mm sieve.

### 3.3. Supercritical Fluid Extraction

Extraction was performed using a laboratory-scale supercritical fluid extraction system (SFE 100 mL, Extratex, Pont-Saint-Vincent, France). The extraction cell was filled with 30 ± 0.5 g of ground berries, placed in the designated location in the extractor, and the parameters of flow rate, pressure, and temperature were set. Each extraction was performed for 2 h, and at the end of the extraction, the extract from the separator was collected in pre-weighed vials and stored at −18 °C until further analysis. At the end of each extraction, the apparatus was cleaned with 20 mL of isopropanol for 20 min. The isopropanol dissolved the extract remaining in the apparatus piping and was evaporated at 40 °C on a rotary evaporator; the mass of the remaining extract was added to the mass of pure extract to give the total mass of extract.

### 3.4. Determination of Fatty Acids

According to the method ISO 12966-2:2017 [58], fatty acid methyl esters (FAME) were prepared from supercritical extracts. They were analyzed using a 6890N gas chromatography (GC) system (Agilent Technologies, Santa Clara, CA, USA) equipped with a DB-23 ((50%-cyanopropyl-methylpolysiloxane) capillary column (60 m × 0.25 mm × 0.25 μm), and combined with a flame ionization detector according to ISO 12966-4:2015 [59]. The flow rate of the carrier gas (helium) was 1.5 mL min^−1^, the split ratio 60:1, the injection volume 1 μL, the temperature of the injector 250 °C, and that of the detector 280 °C. The temperature program was run from 60 to 220 °C at 7 °C min^−1^ and kept at 220 °C for 17 min. The identification of the peaks was carried out by comparing their retention times with a standard mixture containing 37 FAME (C4–C24). 

### 3.5. Determination of Sterols

A modification of the method ISO 12228:2014-1 [60] was used to determine the content and composition of phytosterols in samples of the lipophilic extract of myrtle fruit. The prepared samples were analyzed using an Agilent Technologies 6890N GC equipped with FID and ChemStation B.04.03 software (Agilent Technologies, Santa Clara, CA, USA). The samples were separated using a DB-17 ((50%-phenyl)-methylpolysiloxane) capillary column (30 m × 0.32 mm × 0.25 μm). The analysis was performed under the following conditions: the carrier gas (helium) flow rate was 1.5 mL min^−1^, the split ratio was 13.3:1, the injection volume was 1 μL, the injector temperature was 290 °C, and the detector temperature was 250 °C. The temperature program was run from 180 to 270 °C at 6 °C min^−1^ and then kept at 270 °C for 30 min. Sterol trimethylsilyl esters were identified through available standards and by comparing mass spectra, obtained from a 5973 mass detector (Agilent Technologies, Santa Clara, CA, USA) with a scan range of 30–550 (*m*/*z*), to the NIST17 database and data from the literature [33].

### 3.6. Determination of Volatiles

For sample preparation, 10 µL of supercritical myrtle extract, 100 µL of internal standard (nerol, c = 10.6500 mg mL^−1^), and 890 µL of n-hexane (HPLC purity, 95%) were mixed. GC-MS analysis (Agilent Technologies 6890N Network, Santa Clara, CA, USA) was performed on an inert mass selective detector (Agilent Technologies 5973, Santa Clara, CA, USA) using a HP-5MS (5% phenylmethylsiloxane) capillary column (30 m × 0.25 mm × 0.25 μm). The flow rate of the carrier gas (helium) was 1 mL min^−1^, the split ratio was 100:1, the injection volume was 1 μL, the temperature of the injector was 250 °C, and that of the transfer-line was 280 °C. The temperature program used was as follows: initial oven temperature 60 °C, 60–145 °C at 3 °C min^−1^, and 145–250 °C at 30 °C min^−1^, and retention at 250 °C for 3 min. The volatiles were identified by comparing their retention times and mass spectra with commercial standards and comparing them with the WILEY9, NIST14, and NIST17 databases using MSD ChemStation B.04.03 Software Data Analysis. In addition, their retention index was compared with data from the literature.

### 3.7. Statistical Analysis

The Design-Expert 10.0 software system (Stat-Ease Inc., Minneapolis, MN, USA) was used for experimental design and the statistical processing of data using Box–Behnken (three variables and three factorial levels). The independent variables were temperature (40, 50, and 60 °C), pressure (200, 300, and 400 bar), and flow rate (20, 30, and 40 g min^−1^). Response surface methodology (RSM) was used to optimize the SFE conditions. Extraction yield (%), PUFA content (%), phytosterol yield (mg kg^−1^), and volatile compound yield (mg kg^−1^) were observed as dependent variables. The fit of the model was tested by determining the coefficient of determination (R^2^) and the lack of fit by the F-test. To determine the significance of the model and the influence of individual factors, an analysis of variance (ANOVA) with a confidence level of 95% was performed. Following the analysis of the obtained models, the optimization of scCO_2_ parameters was performed using the desirability method. All analytical determinations were performed with two repetitions, and results are reported as mean values.

## 4. Conclusions

ScCO_2_ extraction is an effective technique for the extraction of lipophilic compounds from myrtle fruits, where yields ranging from 4.75% to 5.92%, with an average value of 5.20%, were obtained by applying different temperature, pressure, and CO_2_ flow rate conditions. In the extracts obtained, 36 fatty acids were identified, with essential cis-linolenic fatty acid being the most abundant (76.83%), followed by palmitic (8.52%) and oleic acid (7.29%). The average yield of total phytosterols was 14,097.92 mg kg^−1^, and seven phytosterols were identified, with β-sitosterol (12,465 mg kg^−1^) being the most abundant, proving that myrtle fruits are a rich source of these compounds. In addition, 13 volatile compounds (9019.64 mg kg^−1^) were identified, of which myrtenyl acetate (4297 mg kg^−1^) was the most abundant. The optimization performed showed that the extraction and PUFA yield are influenced exclusively by temperature, the increase in which also increases the extract yield, while the PUFA yield decreases. The extraction of phytosterols is significantly influenced by the interaction of pressure and flow rate, while the volatile content is influenced by pressure and temperature.

## Figures and Tables

**Table 1:** Box–Behnken experimental design of SFE and extraction yield.

Exp.	X_1_: T (°C)	X_2_: p (bar)	X_3_: Q (g min^−1^)	Yield
1	50	300	30	5.26
2	60	300	20	5.33
3	60	200	30	5.49
4	60	300	40	5.92
5	50	300	30	5.18
6	50	200	20	5.10
7	60	400	30	5.59
8	50	300	30	5.03
9	50	400	20	5.37
10	40	400	30	4.95
11	50	300	30	5.27
12	50	400	40	5.05
13	40	200	30	4.75
14	50	300	30	5.19
15	50	200	40	5.20
16	40	300	20	4.77
17	40	300	40	5.01

**Table 2:** Average composition of fatty acids (%), phytosterols (mg kg^−1^ of fruit), and volatile compounds (mg kg^−1^ fruit).

Fatty Acids (%)		Phytosterols (mg kg^−1^)		Volatiles (mg kg^−1^)	
C11:0	0.6 ± 0.5	Campesterol	238 ± 69	α-Pinene	1280 ± 436
C15:0	0.1 ± 0.1	24-Methylenecholesterol	88 ± 89	β-Pinene	2 ± 3
C16:0	8.5 ± 0.2	Stigmasterol	76 ± 6	Myrcene	1 ± 2
C17:0	0.2 ± 0.1	β-Sitosterol	12,465 ± 1308	α-Phellandrene	35 ± 21
C18:0	3.1 ± 0.8	Δ^5^-Avenasterol	590 ± 71	3-Carene	42 ± 23
C20:0	0.5 ± 0.0	Δ^7^-Sitosterol	166 ± 29	Limonene	238 ± 58
C22:0	0.1 ± 0.2	Citrostadienol	90 ± 58	1,8-Cineole	2183 ± 487
C23:0	0.8 ± 0.7	Total content in extract	13,937 ± 149	Linalool	659 ± 530
C24:0	0.1 ± 0.1	Yield per fruit DW	723 ± 63	α-Terpineol	105 ± 39
C18:1c	7.3 ± 0.3			Myrtenol	23 ± 19
C20:1	0.2 ± 0.1			Carvone	77 ± 20
C18:2t	0.3 ± 0.1			Geraniol	78 ± 26
C18:2c	76.8 ± 1.6			Myrtenyl acetate	4297 ± 917
C18:3n3	0.4 ± 0.1			Total content in extract	9020 ± 2219
C20:5n3	0.2 ± 0.1			Yield per fruit DW	466 ± 102
Σ SFA	14.0 ± 1.5				
Σ MUFA	7.8 ± 0.2				
Σ PUFA	77.9 ± 1.4				

**Table 3:** Results of the statistical analysis of the influence of extraction conditions on the extraction yield, PUFA content, and phytosterols and volatiles yield.

Source of Variation	Extraction Yield		PUFA Content		Phytosterols Yield		Volatiles Yield	
	(%)		(%)		(mg kg^−1^)		(mg kg^−1^)	
	F-Value	*p*-Value	F-Value	*p*-Value	F-Value	*p*-Value	F-Value	*p*-Value
Model	17.22	<0.001 *	6.16	0.008 *	5.03	0.024 *	5.48	0.018 *
Linear								
X_1_	48.40	<0.001 *	16.19	0.001 *	2.78	0.124	11.12	0.013 *
X_2_	1.05	0.324	0.01	0.908	5.31	0.063	0.64	0.449
X_3_	2.22	0.160	2.27	0.156	5.83	0.047 *	4.20	0.080
Quadratic								
X_1_^2^	-	-	-	-	0.99	0.333	20.32	0.003 *
X_2_^2^	-	-	-	-	7.35	0.036 *	7.98	0.026 *
X_3_^2^	-	-	-	-	3.08	0.105	4.11	0.082
Interaction								
X_1_X_2_	-	-	-	-	2.71	0.121	0.20	0.666
X_1_X_3_	-	-	-	-	0.13	0.735	0.45	0.522
X_2_X_3_	-	-	-	-	16.96	0.005 *	0.35	0.574
Lack of fit	2.84	0.164	4.06	0.095	3.25	0.164	0.49	0.710
R^2^	0.799		0.587		0.866		0.876	

X_1_: temperature; X_2_: pressure; X_3_: flow rate. * indicates that the influence of the factor is statistically significant with *p* ≤ 0.05.

**Table 4:** Optimal conditions of SFE.

Factor	Extraction Yield (%)	PUFA Content (%)	Phytosterol Yield (mg kg^−1^)	Volatile Yield (mg kg^−1^)
Optimal value	5.69	79.34	793.48	629.85
Temperature (°C)	60.0	40.2	40.5	49.5
Pressure (bar)	400.0	235.7	225.0	298.0
Flow rate (g min^−1^)	40.0	31.1	32.5	20.3

## Supplementary Materials

The following supporting information can be downloaded at: https://www.mdpi.com/article/10.3390/molecules29081755/s1, Figure S1: Chromatogram of fatty acids in supercritical CO_2_ extract of myrtle fruit; Figure S2: Chromatogram of phytosterols in supercritical CO_2_ extract of myrtle fruit; Figure S3: Chromatogram of volatile compounds in supercritical CO_2_ extract of myrtle fruit.
