# Supplementary material for: Supercritical CO2 Extraction of Fatty Acids, Phytosterols, and Volatiles from Myrtle (Myrtus communis L.) Fruit
Source: Molecules. 2024 Apr 12;29(8):1755. doi: 10.3390/molecules29081755 (PMC11052497; doi:10.3390/molecules29081755)
Supplement: Supplementary file 1 [file molecules-29-01755-s001.zip › molecules-2882923-supplementary.pdf]

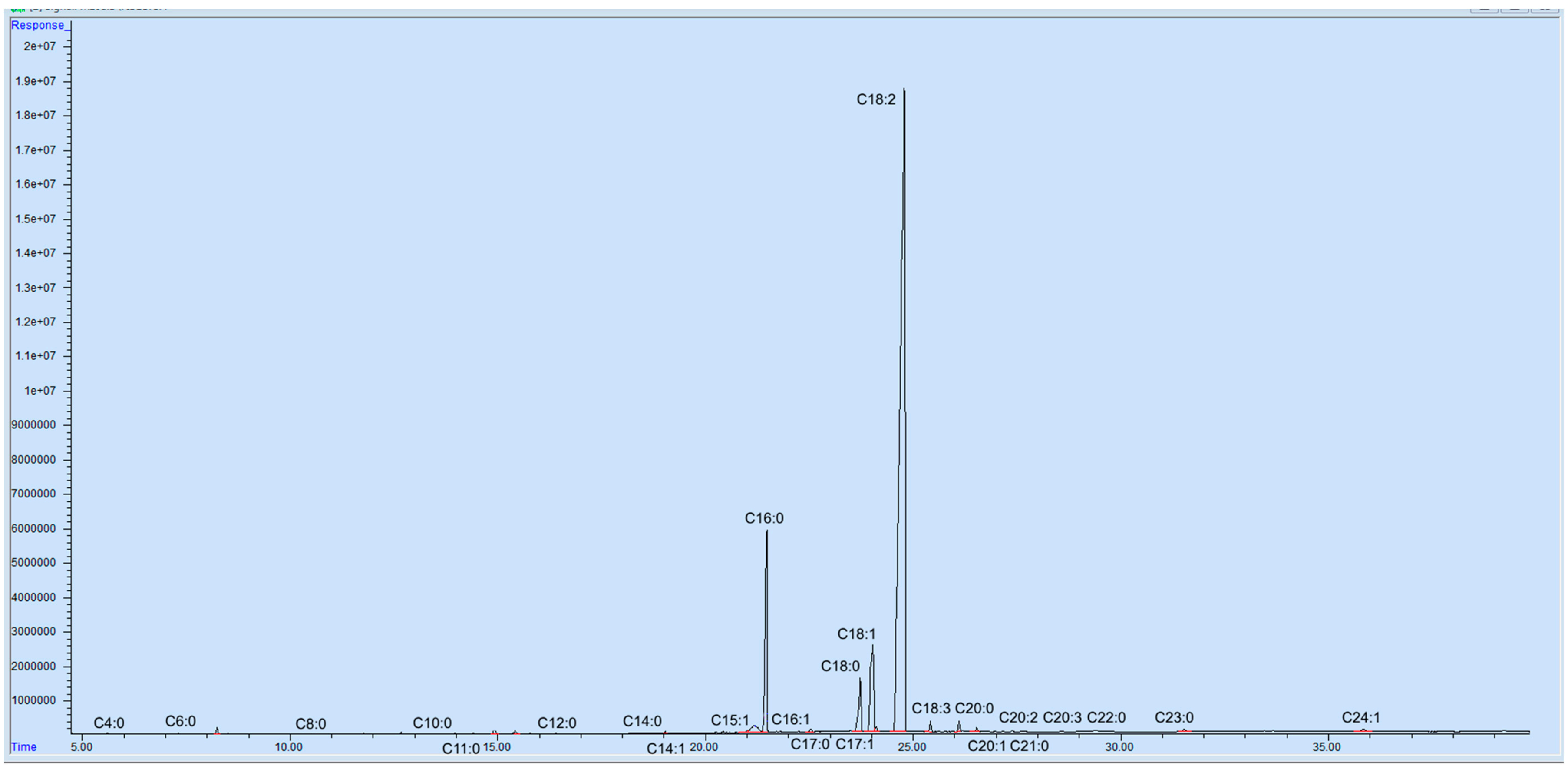

Figure S1. Chromatogram of fatty acids in supercritical CO_2 extract of myrtle fruit

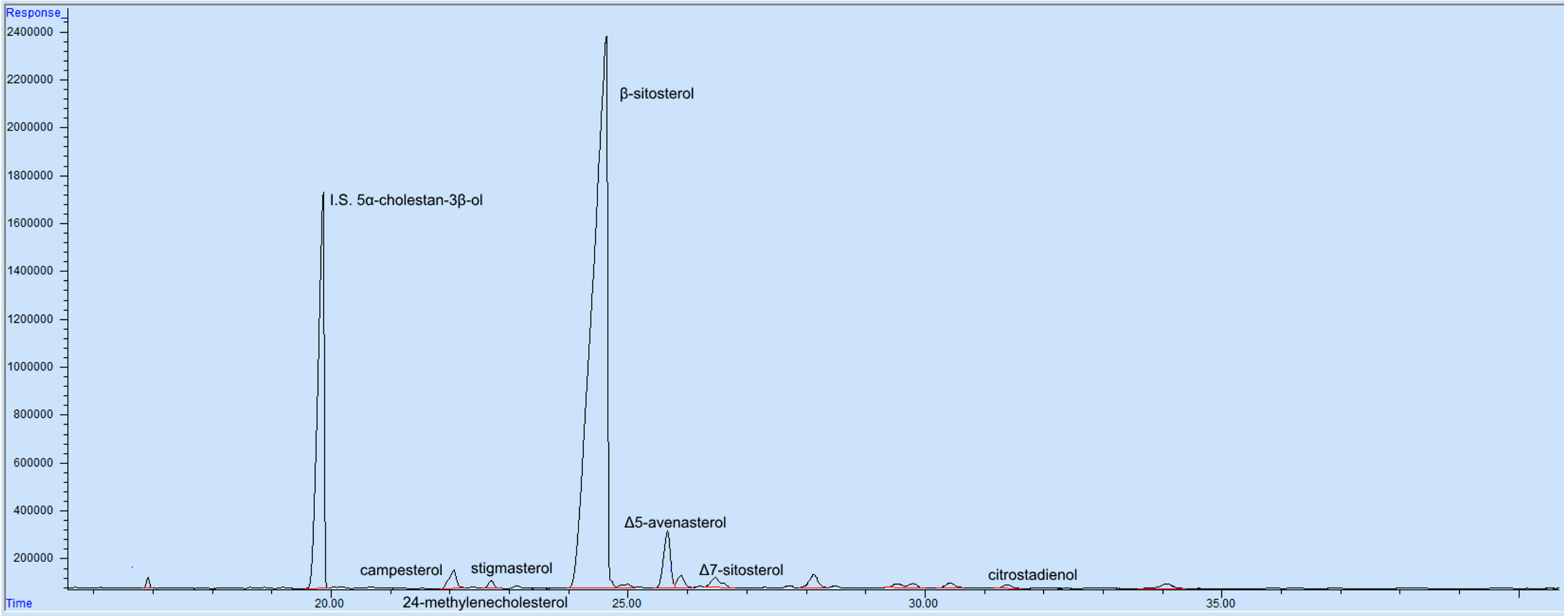

Figure S2. Chromatogram of phytosterols in supercritical CO_2 extract of myrtle fruit

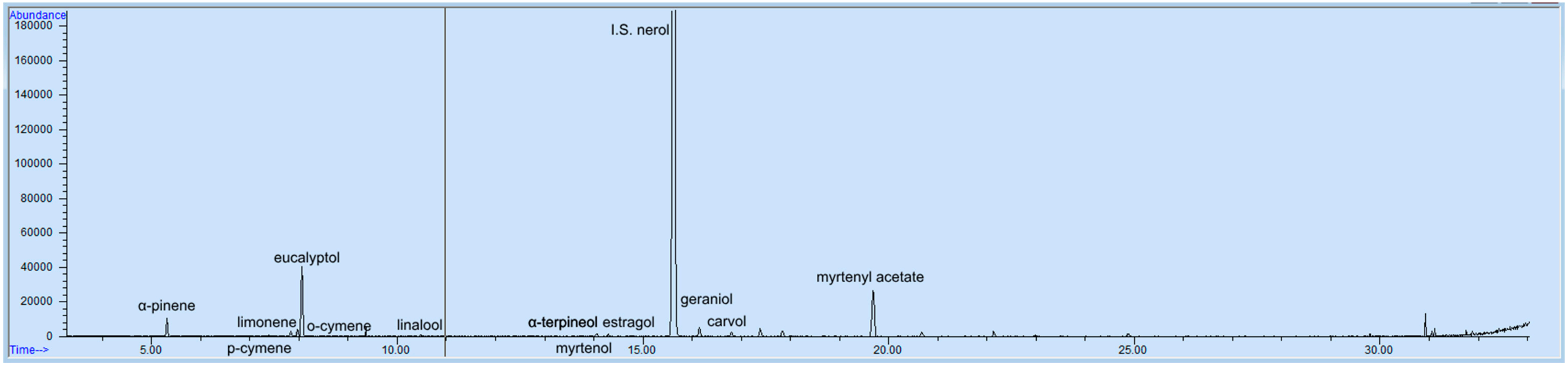

Figure S3. Chromatogram of volatile compounds in supercritical CO_2 extract of myrtle fruit
